# G20 Okayama Health Ministers’ Meeting: Conclusions and commitments

**DOI:** 10.7189/jogh.10.010320

**Published:** 2020-06

**Authors:** Hideaki Nishizawa, Yoshito Nishimura, Hiroshi Matsumura, Hisayo Horiuchi, Toshitaka Higashira, Yosuke Kita, Yasuyuki Sahara, Yasuhiro Suzuki

**Affiliations:** 1 Ministry of Health, Labour and Welfare, Tokyo, Japan; 2 Department of General Medicine, Okayama University Graduate School of Medicine, Dentistry and Pharmaceutical Sciences, Okayama, Japan

Since the initial years of the Group of 20 (G20) Summit, experts have awaited its high-level, concrete commitments to global health [1,2]. The G20 Health Ministers’ Meeting (HMM) began under the German presidency in 2017; Argentina and Japan subsequently established the momentum to retain health as the top priority in G20 countries. During the Japanese presidency in 2019, the third G20 HMM took place in Okayama in October after the G20 Osaka Summit in June, highlighting “the Achievement of Universal Health Coverage (UHC),” “Response to Population Ageing,” and “Management of Health Risk and Health Security including Antimicrobial Resistance (AMR)” as the three main pillars. This report illustrates how the G20 Health Ministers have implemented the contents of the Leaders’ Declaration [3].

## UNIVERSAL HEALTH COVERAGE

There are several key messages in the G20 Okayama Health Ministers’ Declaration [4]. As the meeting was the first important one on global health after the United Nations High-Level Meeting on UHC in September, the Ministers reaffirmed their commitment to achieve UHC by 2030 and agreed on the direction of specific policy measures toward achieving this goal. Ahead of the G20 Summit in June, McBride and colleagues pointed out that G20 health agendas lacked greater commitment to equity [5]. The Ministers expressed their firm determination to advance the health-related sustainable development goals (SDGs) and UHC in the Declaration. Additionally, the importance of building human resources to develop health policies was stressed in the deliverables. Naturally, frontline officers have significant requirements to practically implement high-level messages, and they were verbalized to expedite specific actions. One of the important aspects of the Ministers’ Declaration was the collaboration of the health and finance sectors. As affirmed in the first G20 Joint Session of Ministers of Finance and Health and as pointed out in the World Bank’s report [6], their collaboration is essential to secure the sustainability of health financing. As Dieleman and colleagues mentioned, the G20 has a substantial role in developing assistance for health [7]. While the G20 and international organizations should help developing countries in need, their domestic financing also needs to be strengthened. The G20 Shared Understanding on the Importance of UHC Financing in Developing Countries [8], to which finance and health ministers of G20 affirmed their commitments, identifies several key considerations to strengthen health system towards UHC.

## POPULATION AGEING

Population ageing is a new challenge facing the G20 HMM. Although Japan has always been at the forefront of the issue, it has now become a global agenda. The Declaration highlighted prioritization of active and healthy ageing and the importance of preventive measures to avoid ill-health over one’s life course. The Ministers also stressed the importance of multi-sectoral policy responses to dementia, such as promoting risk reduction and offering inclusive environments, by developing national action plans that have not yet been implemented by all the G20 members. The G20 members are diverse in terms of the pace and stage of population ageing, and the Ministers agreed to continue to share their experiences and play a role in building better policy responses together.

## HEALTH EMERGENCIES AND AMR

Regarding the management of health emergencies, the Ministers agreed that there was still room for enhancing preparedness and responses to global health threats, as indicated by the current Ebola Virus Disease (EVD) outbreak in the Democratic Republic of Congo, despite the substantial improvement in the global health security architecture after the 2014 EVD outbreaks in West Africa. They also agreed to encourage contributions to secure sustainable global financing mechanisms such as the World Health Organization Contingency Fund for Emergencies. Given that it is mostly funded by Germany, Japan, and the United Kingdom, diversifying the donor base is an urgent issue for this essential health emergency countermeasure [9]. The Ministers also agreed to strengthen their efforts to tackle AMR by enhancing stewardship and encouraging investment in the research and development of new antimicrobials.

**Figure:**
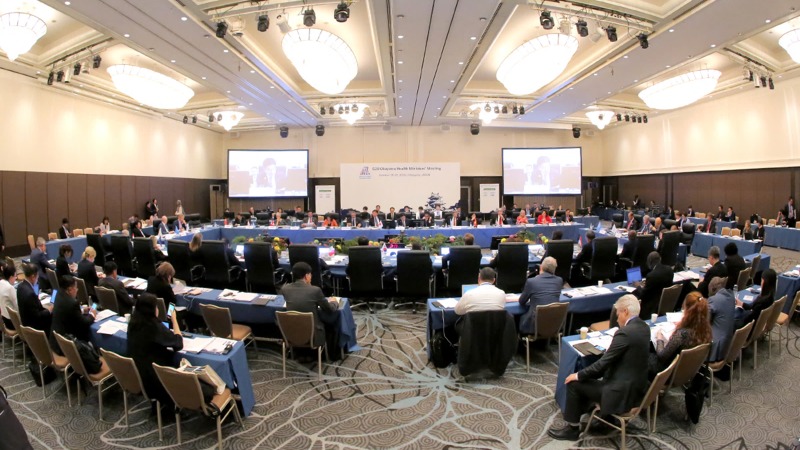
Photo: Health Ministers engaging in discussion to make progress in global health (from the website of the Ministry of Health, Labour and Welfare of Japan, used with permission. https://www.mhlw.go.jp/seisakunitsuite/bunya/hokabunya/kokusai/g20/health/jp/photos_g20okayama.html).

## CONCLUSIONS

The G20 Okayama HMM was a milestone in global health; the Ministers agreed on the direction of policy measures to achieve UHC, presented responses to population ageing as a major global health issue, and strengthened efforts to improve global health risk management and tackle AMR. As indicated in the article published after the G20 Osaka Summit on July 6 [10], we believe that concrete actions by G20 members based on the Declaration are now needed to make substantial progress in global health. In 2020, the Saudi G20 Presidency proposed “Enabling Person-Centered Health Systems” with a focus on value-based health care and digital health solutions as the main pillar of the health agenda [11]. We hope that the next Presidency will accelerate progress in global health, building on the legacy of the previous G20 HMM.
